# Influence of preoperative simulation on the reduction quality and clinical outcomes of open reduction and internal fixation for complex proximal humerus fractures

**DOI:** 10.1186/s12891-023-06348-3

**Published:** 2023-03-30

**Authors:** Rui-Ji Wu, Wei Zhang, Yan-Ze Lin, Zhang-Lu Fang, Kang-Nan Wang, Chang-Xing Wang, Dong-Sheng Yu

**Affiliations:** 1 Department of Orthopedics, Zhejiang Provincial People’s Hospital (Affiliated People’s Hospital, Hangzhou Medical College), Hangzhou, China; 2 The Second Affiliated College of Zhejiang Chinese Medicine University, Hangzhou, China; 3 Department of Orthopedic Surgery, The Second Affiliated Hospital of Zhejiang Chinese Medical University, Hangzhou, China

**Keywords:** Proximal humeral fractures, Open reduction and internal fixation, Preoperative simulation, 3D Printing technology, Computer virtual technology

## Abstract

**Purpose:**

Proximal humerus fractures (PHFs) are common. With the development of locking plates, open reduction and internal fixation (ORIF) of the proximal humerus can provide excellent clinical outcomes. The quality of fracture reduction is crucial in the locking plate fixation of proximal humeral fractures. The purpose of this study was to determine the impact of 3-dimensional (3D) printing technology and computer virtual technology assisted preoperative simulation on the reduction quality and clinical outcomes of 3-part and 4-part proximal humeral fractures.

**Method:**

A retrospective comparative analysis of 3-part and 4-part PHFs undergoing open reduction internal fixation was performed. Patients were divided into 2 groups according to whether computer virtual technology and 3D printed technology were used for preoperative simulation: the simulation group and the conventional group. Operative time, intraoperative bleeding, hospital stay, quality of fracture reduction, Constant scores, American Society for Shoulder and Elbow Surgery (ASES) scores, shoulder range of motion, complications, and revision surgeries were assessed.

**Results:**

This study included 67 patients (58.3%) in the conventional group and 48 patients (41.7%) in the simulation group. The patient demographics and fracture characteristics were comparable in these groups. Compared with the conventional group, the simulation group had shorter operation time and less intraoperative bleeding (P < 0.001, both). Immediate postoperative assessment of fracture reduction showed a higher incidence of greater tuberosity cranialization of < 5 mm, neck-shaft angle of 120° to 150°, and head shaft displacement of < 5 mm in the simulation group. The incidence of good reduction was 2.6 times higher in the simulation group than in the conventional group (95% CI, 1.2–5.8). At the final follow-up, the chance of forward flexion > 120° (OR 5.8, 95% CI 1.8–18.0) and mean constant score of > 65 (OR 3.4, 95% CI 1.5–7.4) was higher in the simulation group than the conventional group, as well as a lower incidence of complications in the simulation group was obtained (OR 0.2, 95% CI 0.1–0.6).

**Conclusions:**

This study identified that preoperative simulation assisted by computer virtual technology and 3D printed technology can improve reduction quality and clinical outcomes in treatment of 3-part and 4-part PHFs.

Proximal humeral fractures (PHFs) are common, accounting for 4–5% of all fractures in adults [1, 2]. In addition, PHFs are the third most common osteoporotic fractures, second only to hip and distal radius fractures [3]. Three-quarters of the patients were older than 60 and women had 2.4 times fracture risk compared with men. [4]. Meanwhile, as the population continues to aging, the incidence of PHFs is likely to continue increasing. Notably, the shoulder joint has the largest range of motion that can perform three-axis motion: adduction and abduction on the coronal axis, flexion and extension on the sagittal axis, and internal and external on the vertical axis. Impaired shoulder mobility, especially in the dominant arm, will greatly reduce the life quality of the patient and sometimes even cause difficulty to live independently. Although conservative treatment was reported with an acceptable outcome for many patients, surgical treatment is still recommend in significantly displaced fractures and young patients required high mobility needs. Traditional ORIF as a common surgical treatment for PHFs required the guidance of 3D-CT and X-ray examination to illustrate the positional relationship of fracture fragments. This technique required high imaging experience during the operation. Meanwhile it is quite difficult to adequately show the shape of the fragments as well as the angles and distances between the fragments. Satisfactory outcome of ORIF of PHFs mainly depends on the quality of the fracture reduction [5, 6]. Thus, more details of the complex fracture should be obtained and organized before operating to formulate accurate surgical strategies which may help the surgeon improve the quality of fracture reduction as well as achieve stable fixation. The computer virtual technology and 3D printing technology established based on CT scans can directly present details and changes in the number, size and location of the fracture fragments [7–10]. In addition, virtual models and 3D printed models can also provide repeatable and interactive virtual surgery, which can help surgeons develop personalized preoperative plans, shorten operative time, reduce intraoperative bleeding and chose appropriate internal fixation instruments [10]. Indeed, there seemed to be little reports aimed to illustrate the effect of preoperative simulation on the reduction quality of complex PHFs.

## Methods

### Study population

This is a retrospective case-control study approved by the Ethics Review Committee of Zhejiang Provincial People’s Hospital (No. QT2022278). From February 2015 to April 2020, 198 patients with PHFs treated with ORIF were identified by searching the hospital database. Inclusion criteria included age ≥ 18 years, 3-part fracture involving the greater tuberosity, 4-part fracture and no previous PHFs. Patients with pathological fractures, open fractures, neuromuscular injuries, ipsilateral upper extremity injuries, lack of correct postoperative radiographs and follow-up less than 24 months were excluded. Of the 198 patients, 62 patients were excluded due to an ineligible fracture type, 3 patients were excluded due to previous PHFs, 4 patients were excluded because of combined with ipsilateral upper extremity injuries, and 14 patients were excluded because the follow-up period was less than 24 months. Thus, 115 patients with complex PHFs were included in this study. Conventional surgery was performed between February 2015 and June 2018. From July 2018 to April 2020, preoperative simulation was performed in all eligible patients with complex PHFs. Patients were divided into 2 groups according to whether the preoperative simulation was performed with the assistance of computer virtual technology and 3D printed technology: the simulation group and the conventional group.

### Preoperative simulation

**Fig. 1:**
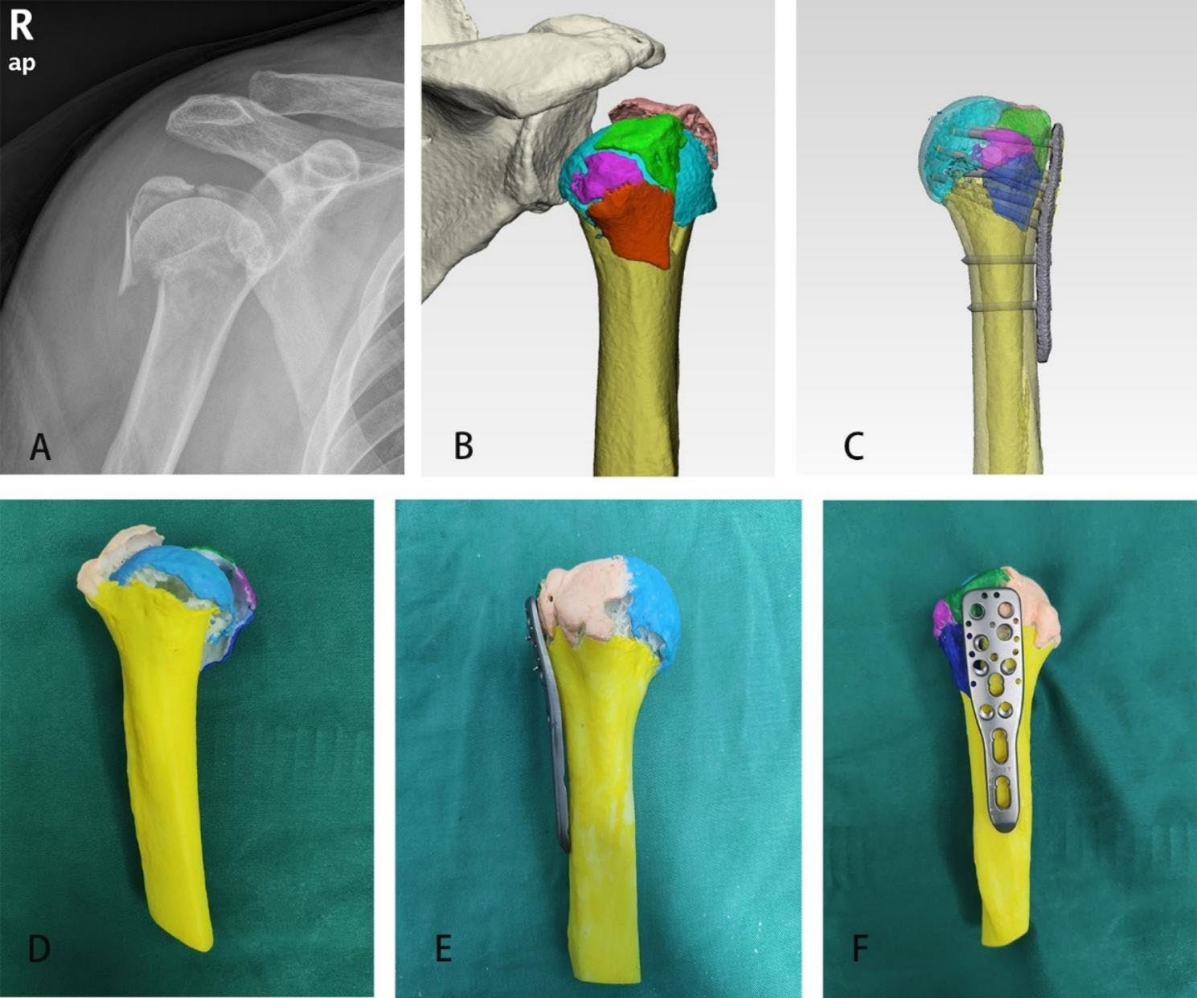
Anterior-posterior view of right shoulder showing proximal humerus fracture (A). 3D reconstructed virtual model of proximal humerus fracture (B). Virtual model for preoperative simulation (C). 3D printed model of proximal humerus fracture (D). 3D printed models for preoperative simulation (E and F)

In the simulation group, the raw 2-dimensional cross-sectional images obtained from a Siemens-defined 128-slice helical CT scanner were transferred to Mimics 21.0 software (MATERIALIZE, Leuven, Belgium) in Digital Imaging and Communications in Medicine (DICOM) format. The original virtual fracture model was established by adjusting the threshold and screening the bone fragments (Fig. 1). The fracture models were then segmented by fracture fragments, marked with different colors to distinguish bone fragments, and imported into Materialise 3-Matic software (MATERIALISE LTD, Leuven, Belgium) in STL format to simulate surgery. The morphology of the fracture line, the shape, size and displacement direction of the fracture fragments and the condition of bone defects was later observed and recorded. Reduction of fractures included correction of head dislocation, reduction of head fragments to the shaft, correction of varus or valgus displacement, restoration of the medial column, and reduction of displaced tuberosity. Subsequently, an appropriate proximal humerus locking plate and screws were chosen followed by the determination of additional medial supports. The surgeon could repeat the simulation operation until a satisfactory reduction was achieved. The final surgical strategy was then validated on a 3D printed model (Fig. 1). The production and operation of a 3D printed model were consists of the following steps: Firstly, the original virtual fracture model was stored in a stereolithography format and subsequently transferred to a 3D printer (Objet Connex350™; Stratasys Ltd., Eden, MN, USA). Secondly, the 3D model is printed according to a 1:1 scale using a medical photosensitive resin material. Finally, the 3D printing model simulates the surgical process again.

In the conventional group, preoperative planning was based on radiographs, CT images, and the surgeon’s experience.

### Operative technique

Patients were prepared in the beach-chair position and all surgical procedures were performed with a deltoid-pectoral approach. All operations were performed by one surgical team leaded by the same operating surgeon as of 2015 who had more than 13 years’ experience in trauma surgery especially in complex fractures around the shoulder. Fracture reduction and internal fixation were performed according to the preoperative plan. The quality of reduction and the positions of plates and screws were verified by intraoperative fluoroscopy.

### Data collection and outcome measures

Patient demographics and fracture characteristics recorded included age, sex, body mass index (BMI), bone mineral density, American Society of Anesthesiologists (ASA) classification, affected and dominant limb, trauma energy, Neer classification, initial neck shaft angle (a 135° neck shaft angle is considered inherent ), dislocation and duration of follow-up. Bone mineral density was measured using dual energy X-ray absorptiometry. Outcome indicators included fracture reduction quality, clinical scores, range of motion, complications, and reoperation rates were recorded. The quality of reduction was determined by assessing standard anteroposterior radiographs within 3 days after surgery. The outcome of reduction quality was evaluated by two orthopedic surgeons both with more than 7 years’ experience in traumatic orthopedics. They were not involved in any operations of this study and were blinded to patient information, grouping information and clinical outcomes.The evaluation criteria of reduction quality included cervical neck-shaft angle displacement ≤ 150° to 120°, head shaft displacement < 5 mm, and greater tuberosity cranialization < 5 mm [5]. If the above three criteria are fulfilled, the reduction quality is considered to be good, otherwise the reduction quality is considered to be malreduction. When there is controversial about the reduction quality, the final conclusion depend on the consensus after re-analysis. Clinical scores include the Shoulder and Elbow Surgeons Standard Shoulder Assessment Scale (ASES) score and Constant score, ranging from 0 to 100, with higher scores for both indicating better shoulder function. The ASES score includes pain (50%) and living function (50%). Constant scores included pain (15%), impact on daily life (20%), range of motion (40%), and strength (25%). The shoulder range of motion includes forward flexion, abduction, external rotation, and internal rotation. External rotation is measured with the elbows at side and flexed 90°. Internal rotation is measured by the level of the spine that the patient’s thumb can reach. Radiological complications include loss of fixation (secondary varus collapse or valgus collapse with or without secondary intra-articular penetrating screws, screw backout, bone plate separation and plate or screw fracture), nonunion, avascular necrosis of the humeral head (with or without intra-articular penetration of screws), greater tuberosity resorption, and subacromial impingement [11].

### Statistical analysis

Statistical analyses were performed using SPSS software (version 20.0; SPSS Inc., Chicago, IL, USA). Kolmogorov-Smirnov test was used to confirm normal distribution of continuous variables before our results were further analyzed. Continuous variable results are reported as mean ± standard deviation. The independent t-tests were used to analyze quantitative data, the Mann-Whitney U-tests were used to compare ranked data, and the chi-square and Fisher exact tests were used to compare categorical information. The level of statistical significance was set at a p value of < 0.05.

## Results

### Demographics

From February 2015 to April 2020 a total of 115 patients with PHFs were included in the study, 67 patients (58.3%) in the conventional group and 48 in the simulation group (41.7%). The mean age of the study population was 64.5 ± 7.8 years (43–86 years) and the mean body mass index was 24.4 ± 3.5 (17.7–36.5), including 91 women (79.1%), 57 patients (49.6%) with dual-energy X-ray T-value ≤-2.5, and 36 patients (31.3%) with ASA score of 3 or 4. Seventy-four patients (64.3%) were involved in low energy trauma mechanisms, usually a simple fall (53/74;71.6%). The most common cause of high energy trauma was a fall from an electric vehicle (17/41;41.5%). The demographic characteristics and fracture characteristics of the patients in both groups were comparable (Table 1). A significant shorter duration of surgery and less intraoperative bleeding were obtained in the simulation group compared to the conventional group: mean duration of surgery of 110.1 ± 26.4 min (conventional) and 82.9 ± 12.8 min (simulation), respectively (P < 0.001), and mean intraoperative bleeding of 149 ± 33.7 ml (conventional) and 112.9 ± 21.2 ml (simulation), respectively (P < 0.001). No significant difference was obtained in duration of hospital stay between the two groups.

**Table 1:** Patient Demographics and Fracture Characteristics

	Conventional Group (n = 67)	Simulation group (n = 48)	P
Age (year)	63.9 ± 7.8	65.2 ± 8.0	0.41
Sex			0.637
Female	52(77.6%)	39(81.2%)	
Male	15(22.4%)	9(18.8%)	
BMI (kg/m^2^)	24.6 ± 3.7	24.0 ± 3.0	0.319
DXA total body T score	-2.2 ± 0.9	-2.3 ± 0.9	0.752
ASA score			0.423
1 or 2	48(71.6%)	31(64.6%)	
3 or 4	29(28.4%)	17(35.4%)	
Dominant arm	32(47.8%)	21(43.8%)	0.672
Trauma energy			0.221
High	27(40.3%)	14(29.2%)	
Low	40(59.7%)	34(70.8%)	
Neer fracture type			0.128
3-Part greater tuberosity	39(58.2%)	21(43.8%)	
4-Part	28(41.8%)	27(56.2%)	
Initial neck shaft angle			0.282
< 120°	18(26.9%)	16(33.3%)	
< 135° to 120°	21(31.3%)	9(18.8%)	
135°-150°	11(16.4%)	13(27.1%)	
> 150°	17(25.4%)	10(20.8%)	
Shoulder dislocation	8(11.9%)	7(14.6%)	0.679

Data: number (%) or mean ± standard deviation

BMI body mass index, DXA dual energy X-ray absorptiometry, ASA American Society for Shoulder and Elbow Surgery

### Reduction quality

The results identified that the simulation group had a higher probability to achieve a good reduction quality compared to the conventional group (Table 2). Immediate postoperative radiographs in the conventional group showed that 50 (74.6%) patients had a neck-shaft angle of 120°-150°, 42 (62.7%) patients had a head-shaft displacement of < 5 mm, and 46 (68.7%) patients had a cranialization of the greater tuberosity of < 5 mm. Malreduction was observed in 33 (49.3%) patients. In the simulation group, 44 (91.7%) patients had a neck-shaft angle of 120°-150°, 39 (81.2%) patients had a head-shaft displacement of < 5 mm, and 43 (89.6%) patients had a cranialization of the greater tuberosity of < 5 mm, and 13 patients (27.1%) had a malreduction. Overall, the chance of patients had a neck-shaft angle of 120°-150°, a head-shaft displacement of < 5 mm and cranialization of the greater tuberosity of < 5 mm were 3.7 (95% CI 1.2–12.0), 2.6 (95% CI 1.1–6.2) and 3.9 (95% CI 1.4–11.3) times higher in the simulation group than in the conventional group, respectively (Table 3). Compared with the conventional group, patients in the simulation group had a 2.6 times higher chance of achieving a good reduction postoperatively (95% CI 1.2–5.8).

**Table 2:** Clinical Outcomes in Different Groups

	Conventional Group (n = 67)	Simulation Group (n = 48)	P
Duration of surgery (min)	110.1 ± 26.4	82.9 ± 12.8	< 0.001
Blood loss volume (ml)	149 ± 33.7	112.9 ± 21.2	< 0.001
Duration of hospital stay (days)	10.8 ± 2.7^*^	11.1 ± 1.8	0.494
Neck-shaft angle			0.02
120°-150°	50 (74.6%)	44 (91.7%)	
< 120° or > 150°	17 (25.4%)	4 (8.3%)	
Head-shaft displacement			0.031
< 5 mm	42 (62.7%)	39 (81.2%)	
> 5 mm	25 (37.3%)	9 (18.8%)	
Greater tuberosity cranialization			0.008
< 5 mm	46 (68.7%)	43 (89.6%)	
> 5 mm	21 (31.3%)	5 (10.4%)	
Overall reduction quality			0.017
Good	34 (50.7%)	35 (72.9%)	
Malreduced	33 (49.3%)	13 (27.1%)	
ASES score	70.7 ± 14.6	79.0 ± 12.0	0.002
Constant score	63.0 ± 12.4	71.1 ± 9.7	< 0.001
Forward flexion	128.3 ± 23.1	145.6 ± 16.1	< 0.001
Abduction	98.6 ± 18.4	117.8 ± 19.0	< 0.001
External rotation	35.8 ± 8.8	46.9 ± 7.0	< 0.001
Complications	21 (31.3%)	4 (8.3%)	0.003

Data: number (%) or mean ± standard deviation

^*^n = 65, 2 patients excluded due to infection

ASES American Society for Shoulder and Elbow Surgery

**Table 3:** Odds ratio of outcome: Simulation group vs. Conventional group

	OR	95% CI	P
Good reduction	2.6	1.2–5.8	0.017
Neck-shaft angle of 120°-150°	3.7	1.2–12.0	0.02
Head-shaft displacement of < 5 mm	2.6	1.1–6.2	0.031
Greater tuberosity cranialization of < 5 mm	3.9	1.4–11.3	0.008
Constant score > 65	3.4	1.5–7.4	0.002
Forward flexion > 120°	5.8	1.8–18.0	< 0.001
Complications	0.2	0.1–0.6	0.003

OR odds ratio, CI confidence interval

### Clinical outcome and range of motion

The simulation group had better forward flexion (133.5 ± 22.9 vs. 115.9 ± 23.8; p < 0.001), abduction (117.8 ± 19.0 vs. 98.6 ± 18.4; p < 0.001) and external rotation (46.9 ± 7 vs. 35.8 ± 8.8; p < 0.001) than the conventional group (Table 2). Patients from the simulation group had a 5.8 (95% CI 1.8–18.0) times higher chance to get a postoperative flexion of > 120° compared with the conventional group.

The Constant score and the ASES score were 63.5 ± 13.5 and 75.8 ± 14.7 for the simulation group and 54.4 ± 13.1 and 66.0 ± 15.4 for the conventional group. The simulation group had a 3.4 (95% CI 1.5–7.4) times higher chance of constant score > 65 than the conventional group (Table 3).

### Complications

At last follow-up, radiological complications were recorded in 25 of 115 (21.7%) patients. The complication rate in the conventional group was 2.6 (95% CI 0.6–11.7) times higher than in the simulation group. In the conventional group, 21 (31.3%) patients developed complications and the most common complication was subacromial impingement (6/21;28.6.6%). Three of six patients were due to upward displacement of the humerus rotation center and the other three were caused by a high plate malposition. Internal fixation failure developed in 5 patients (4 secondary varus collapse and 1 secondary valgus collapse). One of the five patients underwent a reverse shoulder arthroplasty because of a severe varus, and the remaining 4 patients all developed secondary intra-articular screw penetration and had their implants removed at an average of 12 months. Four patients developed avascular necrosis of the humeral head followed by screw penetration within one year after surgery. One of the four patients underwent hemiarthroplasty due to restricted range of motion and persistent pain from shoulder activities, and the orther three patients received implant removal. Two patients had signs of radioactive nonunion between the head and the shaft at 12 months after surgery and subsequently underwent reverse shoulder arthroplasty, while two other patients had resorption of greater tuberosity. In addition, immediate postoperative radiograph demonstrated asymptomatic primary screw penetration in two patients in the conventional group who underwent implant removal after radiological union of the fracture. In the simulation group, no primary screw penetration or radioactive nonunion was observed. Fixation failure occurred in 1 patient, avascular necrosis of the humeral head in 2 patients, and an upward shift of the rotation center in 1 patient without acromial impingement syndrome, all of whom received implant removal only.

## Discussion

Surgical simulation assisted by 3D printed models and virtual models helps the surgeon confirm the details of the fracture, determine the pattern of the fracture line and the number and location of fragments, which might result in a shorter operative time, less intraoperative bleeding and better choice of appropriate internal implants [9, 12]. The purpose of this study was to identify the advantage of preoperative simulation in reduction quality and clinical outcomes in surgical treatment of 3-part and 4-part PHFs compared to conventional operations. In the present study we observed a lower incidence of greater tuberosity cranialization of > 5 mm, head-shaft displacement of > 5 mm and displacement of the neck-shaft angle of > 20° in the simulation group compared to the conventional group. In addition, the simulation group obtained higher constant score and ASES score as well as a lower complication rate compared to the conventional group.

Excellent reduction quality resulted in significantly fewer complications and higher clinical scores [5, 13]. Potential causes of poor clinical outcomes in patients with varus reduction include subacromial impingement, reduced rotator cuff preload, reduced supraspinatus efficiency, and fixation failure. When the varus malreduction was > 20°, the arm elevation forces was significantly increased, and when the varus malreduction reached 45° the efficiency of the supraspinatus was significantly decreased [14]. Fleischhacker et al. analyzed 685 patients and found that mean constant score was significantly associated with the degree of varus (r = -0.23, p < 0.05), for patients with varus < 10°, varus < 20 to 10 and varus < 30°, the mean constant score were 72.5 ± 18.8, 64.7 ± 16.9 and 54.1 ± 19.5, respectively [13]. Varus malreduction increases the risk of secondary screw penetration and screw backout caused by the increased varus torque and the stress on the locking screw due to the increase of lever arm of the rotator cuff. Meanwhile, screw penetration and subacromial impingement are the most common causes of revision surgery.

Several studies have demonstrated that varus malreduction was an important predictor for fixation failure [15–17]. Agudelo et al. found a significant difference in fixation failure rate: the failure rate reached 30.4% when the neck-shaft angle was < 120°, whereas 11% when the neck-shaft angle was > 120° [11]. In our study, 6 patients developed fixation failure, among whom 5 patients had a neck-shaft angle of < 120°. However, Schnetzke et al. found that mild varus (neck-shaft angle of < 120° to 110°) was not associated with a higher complication rate or poor clinical outcome in type C fractures. In contrast, patients with a neck-shaft angle > 150° had lower constant score (38.5 ± 19.9) compared to those with a neck-shaft angle of 130° to 150° (57.5 ± 27.8; p = 0.026). These authors suggested that mild varus could increase the medial contact surface and intrinsic stability under the premise of medial support [5].

Restoration of medial support was critical to prevent fixation failure after surgical treatment in PHFs. Fracture fixation is considered to have medial support when one of the following criteria has been achieved: (1) the medial calcar of the proximal humerus is not comminuted and is anatomically reduced, (2) the shaft is intermediated and impinges on the head fragment, and (3) a calcar screw is placed into the inferomedial quadrant of the proximal humeral head to within 5 mm of the subchondral bone [18, 19]. Krappinger et al. found that the incidence of fixation failure was 6.8% (3/44) in patients with medial support while 43.5% (10/23) in patients without medial support (p < 0.01) [19]. Another previous study also demonstrated that the absence of medial support was a significant predictor of fixation failures [18]. Proximal humerus bone defect and local bone mineral density were also related factors for treatment which is critical to avoid collapse or secondary necrosis [20, 21]. Head-shaft displacement of > 5 mm was also reported as a significant predictor of poorer clinical outcomes (RR,2.8). A mean constant score of 45.8 ± 24.3 was observed in patients with head-shaft displacement of > 5 mm while 64.2 ± 24.7 in patients with head-shaft displacement of < 5 mm (p = 0.001) [5].

In our study, a significant improved reduction quality of the greater tuberosity by preoperative simulation was obtained. The reduction of the greater tuberosity became straightforward with the reduction of the calcar and neck-shaft angle. The incidence of cranial ossification of greater tuberosity cranialization of > 5 mm was 3.9 times higher in the simulation group than in the conventional group.

Schnetzke et al. found that a greater tuberosity cranialization of > 5 mm was a risk factor for a mean constant score% < 50% (RR,3.4; P = 0.009), complications (RR, 3.1) and revision surgery (RR, 2.8) [5]. Patients with a cranialization of the greater tuberosity of > 5 mm might suffer impaired range of motion due to subscapularis impingement. Patients with greater tuberosity cranialization of > 5 mm might experience subscapularis impingement, greater tuberosity nonunion and greater tuberosity resorption resulting in a poor clinical outcome. Similarly, the 2 patients presented with greater tuberosity resorption in our study developed greater tuberosity cranialization of 12.2 and 14.6 mm respectively. Resorption of the greater tuberosity is uncommon after ORIF [22, 23]. Several causes may contribute to resorption of the greater tuberosity: devascularization due to fracture or surgical operation, malreduction of the greater tuberosity and secondary displacement of the fracture. A higher rate of resorption was found in PHFs patients with smaller fragments, more fragments and decreased bone density. In addition, the use of an intramedullary fibular strut was an independent factor for resorption of large nodules (OR, 4.5; p = 0.018). When intramedullary fibular strut was inserted into the epiphysis, the strut grafts may disrupt the intraosseous blood supply and thus lead to resorption of the greater tuberosity [24].

Preoperative simulation with the aid of 3D printed models and virtual models helps surgeons observe and understand the characteristics of fractures directly. Although preoperative simulation requires more time and effort in the preoperative period, the effect is significant in reducing operative time and intraoperative bleeding as well as improving reduction quality.

Nevertheless, some limitations of our study should be noted. As a retrospective study, selection bias and limitations in data collection were difficult to avoid. Some patients lost follow-up or could only have telephone follow-up because of address changes. Thus, we only included patients who were followed up for more than 2 years. The surgeon’s experience played a crucial role in reduction quality and clinical outcomes and different surgeons or surgical teams might cause bias. Hence, the cases involved in this study were all from one surgical team and all the operations were performed by the same operating surgeon from this group to avoid the bias derived from different surgeons or other members. Actually, the operating surgeon already had proven ORIF techniques for PHFs at the beginning of our study. However, the surgical operation process might gradually become more fluent after the surgeon and the team were familiar with the preoperative simulation. However, this does not affect the correctness of our conclusions due to the technical improvement brought by the introduction of simulation methods. Therefore, further randomized prospective studies would be necessary to reduce the limitations.

## Data Availability

The datasets used or analysed during the current study are available from the corresponding author on reasonable request.

## Abbreviations

PHFs: Proximal humerus fractures
ORIF: Open reduction and internal fixation
3D: 3-dimensional
ASES: American Society for Shoulder and Elbow Surgery
CT: Computed tomography
DICOM: Digital Imaging and Communications in Medicine
BMI: Body mass index
ASA: American Society of Anesthesiologists
